# GPx1 deficiency confers increased susceptibility to ferroptosis in macrophages from individuals with active Crohn’s disease

**DOI:** 10.1038/s41419-024-07289-y

**Published:** 2024-12-18

**Authors:** James A. Sousa, Blanca E. Callejas, Arthur Wang, Eve Higgins, Aydin Herik, Natalie Andonian, Munazza Yousuf, Pina Colarusso, Maitreyi Raman, Derek M. McKay

**Affiliations:** 1 Gastrointestinal Research Group, Inflammation Research Network, Department of Physiology and Pharmacology, Calvin, Phoebe and Joan Snyder Institute for Chronic Diseases, Cumming School of Medicine, University of Calgary, Calgary, Alberta Canada; 2 Live Cell Imaging Laboratory, Department of Physiology and Pharmacology, Calvin, Phoebe and Joan Snyder Institute for Chronic Diseases, Cumming School of Medicine, University of Calgary, Calgary, Canada; 3 Department of Medicine, Cumming School of Medicine, University of Calgary, Calgary, Canada; 4 Department of Community Health Science, Cumming School of Medicine, University of Calgary, Calgary, Canada

**Keywords:** Crohn's disease, Chronic inflammation, Monocytes and macrophages, Cell death, Cell death and immune response

## Abstract

Intestinal cell death is a defining feature of Crohn’s disease (CD), a major form of inflammatory bowel disease. The focus on this aspect of enteric inflammation has mainly been on epithelial cells, while other cell types such as stromal and myeloid cells have received less attention. Hypothesising that decreased macrophage viability in an oxidative environment could be a contributing factor to the pathophysiology of CD, we found that monocyte-derived macrophages from individuals with active CD (but not those in clinical disease remission) have increased sensitivity to cell death induced by H_2_O_2_. Molecular biology and pharmacological studies ruled out apoptosis and necroptosis, while increased lipid peroxidation and surface expression of the transferrin receptor implicated ferroptosis as the mechanism of the H_2_O_2_-induced cell death: this was supported by suppression of H_2_O_2_-cytotoxicity by liproxstatin-1, a pharmacological inhibitor of ferroptosis. Selenoproteins are important antioxidants, and selenium deficiency can be a feature of CD. Despite normal dietary intake of selenium, monocyte-derived macrophages and intestinal macrophages in individuals with CD had decreased protein and/or mRNA expression of the selenoprotein, glutathione peroxidase (GPx)-1. Knockdown of GPx1 in macrophages from healthy volunteers resulted in increased H_2_O_2_-induced cell death reminiscent of that observed with macrophages from CD. In summary, monocyte-derived macrophages from individuals with CD have increased susceptibility to H_2_O_2_-induced ferroptosis cell death, that may be facilitated, at least in part, by reduced expression of the antioxidant GPx1. We suggest that reduced GPx1 in monocytes recruited to the gut and intestinal macrophages renders these cells vulnerable to reactive oxygen species-evoked ferroptosis cell death and that unraveling the participation of this pathway in Crohn’s disease may reveal novel therapeutic approaches to this chronic condition.

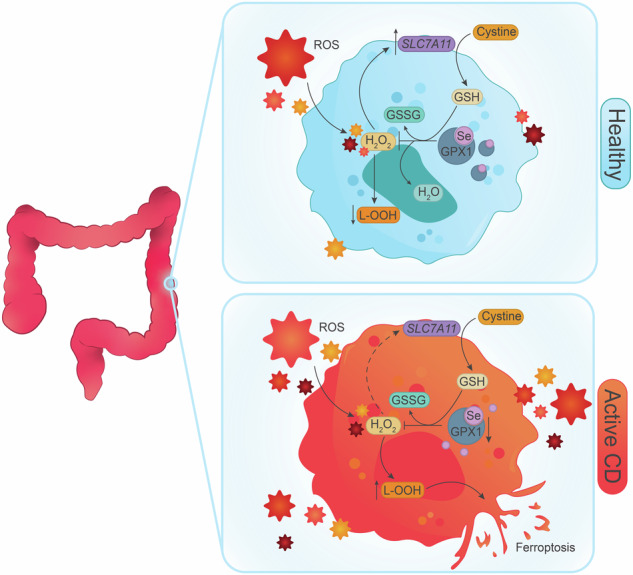

## Introduction

Crohn’s disease (CD), a form of inflammatory bowel disease (IBD), is characterized by inflammation and ulceration in the gastrointestinal tract. While the aetiology of CD remains elusive and disease pathophysiology continues to be defined, the disruption of enteric homeostasis, increased oxidative stress and an imbalance between inflammation and tissue repair have been well characterized in IBD. Macrophages are important regulators of enteric homeostasis through their ability to sample their environment and initiate an immune response, while also participating in tissue repair and wound healing [1, 2]. The gut contains two main populations of macrophages—tissue resident macrophages and infiltrating macrophages [2]. However, in IBD the balance between these two populations is disrupted, with a reduction in tissue resident macrophages and an increase in infiltrating macrophages, which have a more pro-inflammatory phenotype [3–5]. The exact mechanism resulting in this imbalance of populations is not fully understood but dysregulated cell death may play a role [3].

Increased cell death contributes to the pathophysiology of CD and occurs through non-programmed (i.e., necrosis) and programmed cell death pathways (i.e., apoptosis) [6]. To date, the focus has been on the viability of epithelial cells, given that their loss would lead to breaches in the epithelial barrier allowing antigen and pathogens entry to the mucosa. Further, epithelial apoptosis, pyroptosis, and ferroptosis have been described in IBD [6–8]. By contrast, the viability of other intestinal cells such as stromal cells and macrophages in the context of inflammation has received less attention. Analyses of macrophage viability in IBD are limited. Palmer et al. found that monocyte-derived macrophages from patients with CD were less susceptible to cell death induced by phorbol-12-myristate-13-acetate and speculated that this could result in the prolonged presence of pro-inflammatory macrophages in the gut and incomplete resolution of inflammation [9]. In the dextran sodium sulfate (DSS) mouse model of colitis, increased macrophage ferroptosis (an iron-dependent form of cell death characterized by excessive lipid peroxidation [10, 11]) has been described in the colon as a consequence of and contributor to a pro-inflammatory milieu [12]. Despite a limited amount of data, it is reasonable to postulate that dysregulated control of macrophage survival in an inflamed environment could exaggerate inflammation, and this could be relevant to the initiation or progression of CD. In addition, there is increasing interest in the macrophage as a therapeutic target in IBD [13], and yet little is known about their viability and susceptibility to pro-death stimuli.

There is increasing evidence indicating that diet plays a role in the development and treatment of IBD [14]: how diet affects cell death is an emerging area of study. For instance, probiotics, polyphenols, and zinc can protect against increased epithelial cell death in IBD [15]. Other micronutrients such as selenium—a common deficiency in individuals with IBD [16, 17]—is also important for cell survival. For example, when cultured in selenium-deficient conditions, Jurkat cells (a human T cell model) exhibit increased spontaneous cell death [18], due to decreased expression of selenium-dependent antioxidant enzymes (i.e., selenoproteins) such as glutathione peroxidases (GPx) and thioredoxin reductases leading to excessive oxidative stress and eventually cell death. Furthermore, by reducing lipid peroxidation, selenoproteins (e.g., GPx4) inhibit ferroptotic cell death [19].

Therefore, the current study sought to determine if there is any relationship between macrophage viability and selenium status/selenoproteins in IBD. Comparing monocyte-derived macrophages from individuals with CD and matched healthy volunteers, we find that cells from CD are more susceptible to oxidative-stress induced cell death (mimicked by exposure to H_2_O_2_) that occurred via ferroptosis. Furthermore, this was associated with reduced macrophage expression of the selenoprotein, GPx1, despite sufficient selenium intake in this cohort of patients and normal levels of biomarkers indicative of sufficient selenium status.

## Materials and methods

### Human monocyte-derived macrophages

Approximately 40 mL of blood were collected via venipuncture from healthy (no known chronic or acute conditions; not taking medication) individuals (*n* = 26; ages 18–57, 54% female) and individuals with Crohn’s disease in clinical remission or with mild-to-moderate clinical disease activity (*n* = 20 active and 16 remission; ages 19–69, 44% female) recruited through the out-patient IBD clinic at the Foothills Medical Center (Calgary, AB) under ethics protocols: REB15-1270, REB19-0402 and REB 20-1534. Informed consent was obtained prior to study participation. The treating gastroenterologist referred eligible patients based on the criteria in Supplementary Tables 1 and 2. Supplementary Table 3 provides additional patient demographics.

Peripheral blood mononuclear cells were isolated by Ficoll® density gradient centrifugation and monocytes were isolated by plastic adherence by culturing 1 × 10^6^ cells/mL for 2 h in serum-free media at 37 °C [20]. Non-adherent cells were then removed, and the adherent cells were differentiated into macrophages using recombinant good manufacturing practice (GMP) macrophage-colony stimulating factor (M-CSF: 10 ng/mL; R&D Systems, Minneapolis, MN) in RPMI-1640 (Gibco, Detroit, MI) medium supplemented with 2% penicillin/streptomycin (Gibco), 20 mM HEPES (Gibco) and 5% pooled normal human heat-inactivated and sterile-filtered AB serum (Innovative Research, Novi, MI) for 7 days, with a media change on day 5 with fresh M-CSF (10 ng/mL). For macrophage polarization experiments, cells were then treated with either human recombinant IL-4 (10 ng/mL, Cedarlane Labs, Burlington, ON) or IFN-γ (10 ng/mL, eBioscience, San Diego, CA) for 48 h.

### Lactate dehydrogenase (LDH) detection

Macrophages were seeded in a 96-well plate (2.5 × 10^4^ per well in 100 μL) and incubated at 37 °C and 5% CO_2_ overnight to allow adherence. Cells were then treated (duplicate wells/treatment) with stuarosporine (1 μM) for 24 h [21] or H_2_O_2_ (100–2500 μM, Sigma-Aldrich) for 2 h at 37 °C ± a 30 min pre-treatment with inhibitors of cell death: liproxstatin (LPX, 10 µM, Sigma-Aldrich) and necrostatin-1 (25 µM) [22]. Macrophage viability was determined by release of LDH into the culture medium as measured by the CyQuant^TM^ Lactate Dehydrogenase assay kit (#C20300, Invitrogen, Eugene, OR) following the manufacturers’ protocol.

### SYTOX cell death assay

Macrophages were seeded in a 96-well plate (2.5 × 10^4^ cells per well in 100 μL) and incubated at 37 °C and 5% CO_2_ overnight to allow adherence. Next, 1 μM of SYTOX orange (Thermo Fisher Scientific, Rochester, NY) and 500 μM H_2_O_2_ were added to each well. Cells were imaged using the CELLCYTE (Cytena, Bellevue, WA) with the 10X objective (Exposure: 200 ms, Gain: 2 dB) hourly for 12 h. The numbers of SYTOX-positive dead cells were counted in two randomly chosen fields of view.

### Immunoblotting

Total protein was extracted from 5 × 10^5^ macrophages and quantified with the Bradford assay (Bio-Rad). Twenty μg of protein were run on 12% polyacrylamide gels containing 0.1% sodium dodecyl sulfate (SDS) at 80 V for 2 h at room temperature (RT). Next, proteins were transferred to a 0.2 μM PVDF membrane (Bio-Rad) for 16 h at 30 V in transfer buffer (25 mM Tris base, 192 mM glycine, 20% vol./vol. methanol) at 4 °C. The membrane was first blocked in 5% (wt./vol.) skim milk powder (Lab M Limited, Heywood, UK) in tris-buffered saline with 0.1% tween20 (TBST) for 1 h at RT, then was probed with the primary antibody (concentrations and manufacturers in Supplementary Table 4) diluted in TBST with 5% BSA for 16 h at 4 °C on a rocker. The membrane was washed then probed with the appropriate species-matched secondary horseradish peroxidase conjugated antibody (0.4 mg/mL diluted 1:2500 mouse anti-rabbit IgG (sc-2357, Santa Cruz Biotechnology, Dallas, TX) or 0.4 mg/mL diluted 1:2000 goat anti-mouse IgG (sc-2031)) diluted in 5% skim milk powder in TBST for 1 h at RT on a rocker. The membrane was then washed again three times, and the protein bands visualized using Bio-Rad Clarity Western Enhanced Chemiluminescence substrate on a Bio-Rad Chemidoc Imaging System. Densitometry analysis was performed using ImageJ software [23].

### RNA isolation and qPCR

RNA was collected from 1.25 × 10^5^ macrophages using the RNeasy Mini Kit (#74104 Qiagen) as per the manufacturer’s protocol. Complementary deoxyribonucleic acid (cDNA) was synthesized from 0.1 µg of RNA sample using the Quantabio qScript^TM^ cDNA synthesis kit (95047-025, Quantabio, Beverly, MA). For quantitative real-time polymerase chain reaction (qPCR), samples were assayed in duplicate using the SsoAdvanced Universal SYBR Green Supermix (Bio-Rad, Hercules, CA). Primers are listed in Supplementary Table 5. For each reaction, 0.5 μg of cDNA and 0.4 μM forward/reverse primer mix was used. For normalization and absolute gene mRNA expression, *18s rRNA* was used as a housekeeping gene. Relative quantification was assessed using the ∆∆C_T_ method [24].

### ELISA

The concentrations of IL-1β and IL-18 in cell culture media were measured by commercial ELISAs (DY201 and DY318: R&D Systems) following the manufactures’ instructions. Samples were diluted 1:1 or 1:4 for IL-1β and IL-18, respectively, with reagent diluent. The concentration in the supernatant was determined utilizing a standard curve ranging from 4–250 pg/mL and 12–750 pg/mL for IL-1β and IL-18, respectively.

### Lipid peroxidation

Lipid peroxidation after exposure to H_2_O_2_ was assessed using the oxidation-sensitive lipid peroxidation probe Bodipy-C11 (#D3861, Invitrogen) [25]. Macrophages (6.25 × 10^4^ cells in 250 µL) were added to each well of an 8-chamber #1.5 cover glass chamber (Thermo Fisher Scientific). Cells were stained with 2 µg/mL Bodipy-C11 (30 min, at 37 °C and 5% CO_2_) and then left untreated or stimulated with H_2_O_2_ (500 µM) ± the ferroptosis inhibitor, LPX (10 µM). (1S,3R)-Ras Selective Lethal 3 (RSL3; Caymen Chemical Co., Ann Arbor, MI) was used to induce ferroptosis as a positive control (1 µM, 2 h at 37 °C) [26]. After treatment, cells were imaged using the Nikon A1R confocal laser-scanning microscope (Nikon Canada, Mississauga, ON) using Nikon Elements acquisition software (version 5.42.03). Oxidized Bodipy-C11 was excited with a 488 nm laser and emission was imaged at 500–550 nm. Reduced Bopidy-C11 was excited with a 561 nm laser and emission was imaged at 570–620 nm. The field of view was randomly selected, and an image was taken (resonant scanning mode, 20x objective, numerical aperture 0.75, pinhole 20.42 μm, pixel size in x,y 0.63 μm). Mean intensity of the oxidized and reduced forms of Bodipy-C11 was determined on a cell basis by thresholding using ImageJ software and the ratio determined the amount of lipid peroxidation [27].

### Single-cell RNA analyses

For the analysis of selenoprotein gene expression, a single-cell RNA seq (scRNAseq) publicly available dataset of immune cells from healthy individuals and patients with CD (*n* = 71) was reanalyzed (SCP1884) [28]. Using the repository authors’ annotations, macrophage subsets (Supplementary Table 6) were combined for downstream analysis in Seurat v5. The *HarmonyIntegration* setting was used to integrate samples within the macrophage object. Unsupervised clustering was then performed on the integrated data, using the Louvain algorithm, and macrophage subsets were annotated based upon the repository author’s original macrophage annotations. Differential expression analysis was performed to compare macrophages from patients to those from healthy individuals, in addition to comparing macrophages from inflamed and non-inflamed biopsies to healthy individuals. The list of differentially expressed genes was uploaded to Ingenuity Pathway Analysis (QIAGEN IPA), and core analysis yielded a list of canonical pathways, of which the top 10 upregulated and downregulated pathways were visualized on bar plots using ggplot v3.5.0 [29].

### Immunohistochemistry and immunofluorescence

Macrophages (6.5 × 10^3^ cells/well) were seeded in an 8-chamber #1.5 cover glass chamber (Lab-Tek II 155409, Thermo Fisher Scientific) and were allowed to adhere overnight at 37 °C. Cells were then fixed with 4% formaldehyde for 10 min at 37 °C followed by permeabilization with 0.3% Triton-X100 (Sigma-Aldrich, St. Louis, MO) for 10 min at RT. Cells were incubated with a mouse monoclonal antibody targeting the human transferrin receptor (Clone H68.4, #136800, Invitrogen, Eugene, OR) diluted to a final concentration of 2 µg/mL in PBS overnight at 4 °C. The next day, cells were washed with PBS and incubated with goat anti-mouse IgG AlexaFluor 488 (2 μg/mL, A11029, Invitrogen) antibody diluted 1:1000 in PBS for 2 h at RT. Finally, cells were stained with DAPI for 5 min at RT (100 pg/mL, Thermo Fisher Scientific).

For immunohistochemistry, colonic biopsy tissue was collected from individuals with Crohn’s disease undergoing routine colonoscopy and volunteers (controls) undergoing colon cancer screening (ethics ID REB24827). Biopsies were determined to be from areas of active or inactive inflammation by the attending gastroenterologist at the time of colonoscopy. Tissues were fixed with 4% formaldehyde, embedded in paraffin, and 7 μm sections were taken. Antigen retrieval was performed in antigen retrieval buffer (ab93678, Abcam, Eugene, OR) by bringing the solution to a boil by microwaving (1 min) and allowing to cool to RT. Following permeabilization (0.2% Triton-X100 in PBS, 10 min, RT), endogenous peroxidases were blocked by a 10 min incubation in a 3% H_2_O_2_ solution, followed by a 1 h RT incubation in PBS with 2% bovine serum albumin (BSA, Amresco, Solon, OH). Sections were then incubated with primary antibody (rabbit anti-human glutathione peroxidase (GPx)-1, 1 mg/mL diluted 1:250, ab22604; rabbit anti-human GPx4, 0.485 mg/mL diluted 1:50, ab125066; mouse anti-human 4-hydroxynonenal (4-HNE), 1 mg/mL diluted 1:500, MA527570) for 16 h at 4 °C. Subsequently, slides were washed 3X with PBS (10 min, RT) and incubated with the secondary horseradish peroxidase conjugated antibody (mouse anti-rabbit IgG, 0.4 mg/mL diluted 1:500, sc-2357 or goat anti-mouse IgG, 0.4 mg/mL diluted 1:500, sc-2031) for 2 h at RT, followed by three PBS washes and treated with the 3,3′-diaminobenzidine substrate (ab64238, Abcam) for 10 min. Tissue sections were then counterstained with hematoxylin and eosin before dehydrating and cover-slipped in Permount™ mounting media (SP15-100, Fisher Scientific, High River, AB). Random images were captured from blinded/coded slides on an Olympus BX41 microscope (Evident Canada, Montreal, QC) equipped with a color camera (Olympus DP10, cellSens Standard 2.3). Semi-quantitative analysis was performed as previously described [30]. Intensity of DAB staining was quantified and normalized by the number of nuclei present in each image.

### Selenium status

As biochemical markers of selenium status, serum GPx activity (ab102530, Abcam) and SelenoP concentrations (CSB-EL021018HU, CusaBio) were determined using commercial assays and following manufacturers’ protocols. To assess daily selenium intake, two non-consecutive 24 h food recalls were performed using the ASA24® Dietary Assessment Tool (National Cancer Institute) [31]. The information collected was used to estimate daily selenium intake.

### siRNA knockdown

Macrophages (2.5 × 10^5^ cells/mL) were seeded for 24 h and then were transfected with 10 nM of small interfering RNA (siRNA, GPx1 s804, s805, Thermo Fisher Scientific) or a negative control (Silencer Select siRNA Negative control 4394803, Thermo Fisher Scientific) following the manufacturer’s instructions. Protein knockdown was assessed by immunoblotting and qPCR for GPx1. Experiments on siRNA-treated cells were performed 48 h post-transfection.

### Statistical analysis

All data are represented as means ± standard error of the mean (SEM) and *p* ≤ 0.05 was accepted a statistically significant difference. For violin plots, the dashed lines represent the median and the dotted lines the quartiles. Parametric data were analyzed with either a Student’s t-test or a one-way ANOVA with Tukey’s post-test if there were more than two groups. For normalized or non-parametric data, a Wilcoxon signed-rank test, or a Kruskal–Wallis test was used followed with Dunn’s test for group comparisons. GraphPad Prism 10.0.0 software (GraphPad Software, La Jolla, CA) was used to produce graphs and perform the statistical analyses.

## Results

### Monocyte-derived macrophages from Crohn’s disease display increased sensitivity to H_2_O_2_ induced ferroptosis

**Fig. 1:**
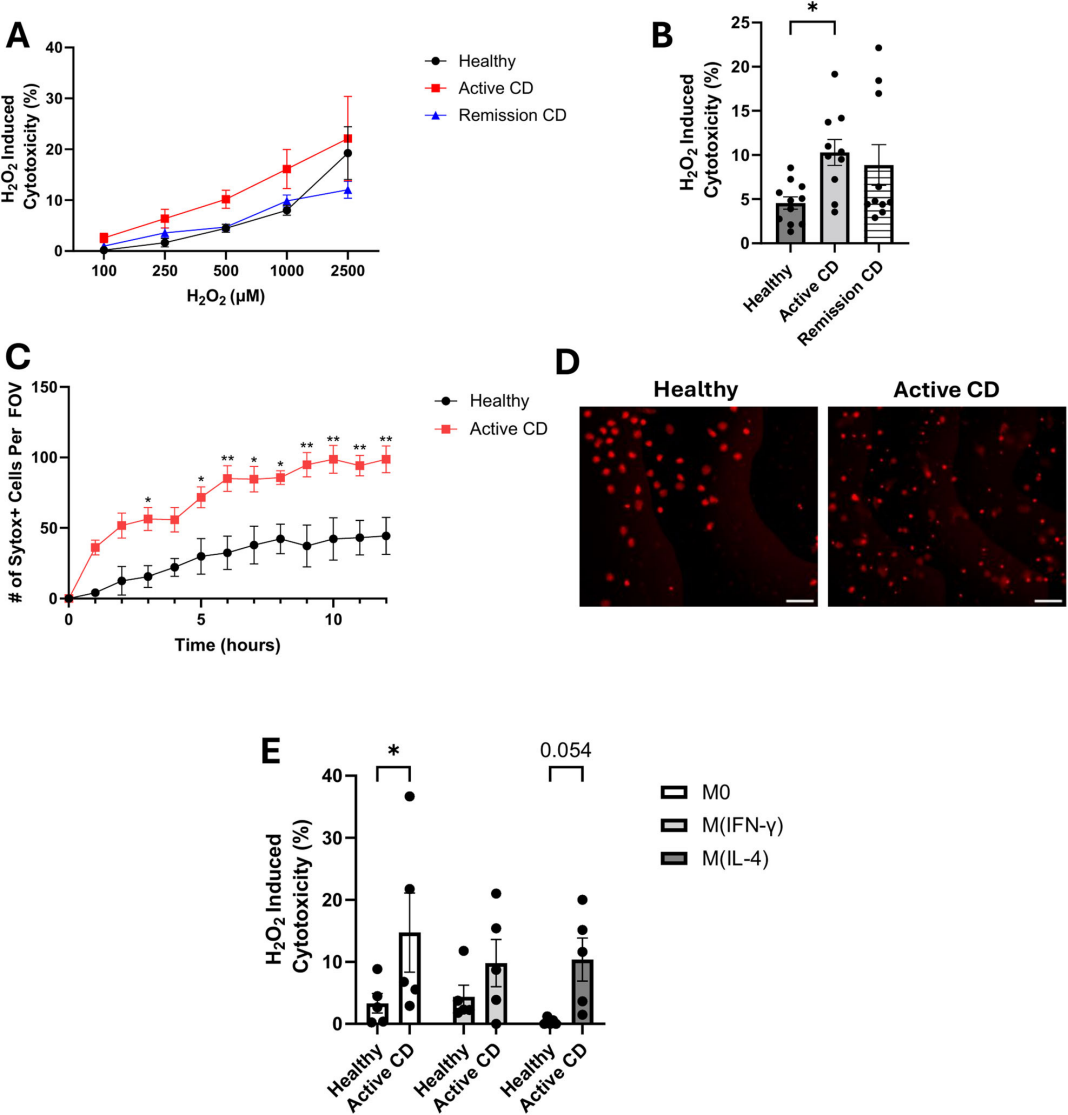
Macrophages from patients with active CD are more susceptible to H_2_O_2_-induced cytotoxicity. Monocyte-derived macrophages (2.5 × 10^4^ cells) were treated with H_2_O_2,_ and cell death was assessed using the LDH assay. Macrophages derived from patients with active CD are more susceptible to H_2_O_2_-induced cytotoxicity (**A**) and this difference is statistically significant at 500 µM (**B**). **C** A time course of H_2_O_2_-induced cell death was performed using SYTOX orange (1 µM) to detect dead cells. H_2_O_2_ induced more cell death in macrophages derived from patients with active CD compared with healthy macrophages as indicated by the number of SYTOX-positive cells. *N* = 3 per group. **D** Representative images from the 12 h time point. Scale bar = 100 µm. **E** Macrophages were treated with either IFN-γ (10 ng/mL) or IL-4 (10 ng/mL) for 48 h then cytotoxicity was induced using H_2_O_2_ (500 µM, 2 h). Data are means ± SEM (*p < 0.05, **p < 0.005).

When retrieving macrophages from plastic culture dishes through trypsinization we see a decreased recovery of macrophages from patients with active CD compared to healthy controls (Table 1). This suggests decreased viability and increased sensitivity to protease-induced damage and cell death. To further explore macrophage viability in patients with CD (both in remission and with active disease), staurosporine (protein kinase inhibitor and driver of apoptosis) and H_2_O_2_ (mimics oxidative stress in an inflamed environment) was used to induce cell death. Treatment with staurosporine (1 µM, 24 h) showed no difference in cell death between groups (control (*n* = 6) 4.8% ± 1.1% vs active CD (*n* = 5) 5.33 ± 0.7% over control non-treated macrophages, *p* = 0.7). However, when macrophages from healthy donors and patients with CD were treated with different concentrations of H_2_O_2_ for 2 h, there was a difference in cytotoxicity as assessed by the release of LDH. At all concentrations tested, H_2_O_2_-induced greater cytotoxicity in macrophages from individuals with active CD compared to healthy controls, with the difference being statistically significant at 500 µM H_2_O_2_ (Fig. 1A, B). This increased sensitivity to H_2_O_2_ (500 µM) was also seen in the SYTOX assay at multiple time points (Fig. 1C, D); in addition, polarization to an M(IFNγ) or M(IL4) phenotype did not provide substantial protection to the CD macrophages from H_2_O_2_-cytoxocity (Fig. 1E).

**Table 1:** Macrophage recovery from plastic culture dishes.

Group	Recovery yield (mean % ± SEM)	*P*-Value
Healthy (*n* = 12)	80.6 ± 3.7	**0.0002**
Active CD (*n* = 12)	45.8 ± 6.7	

Bold values identify statistical significance.

**Fig. 2:**
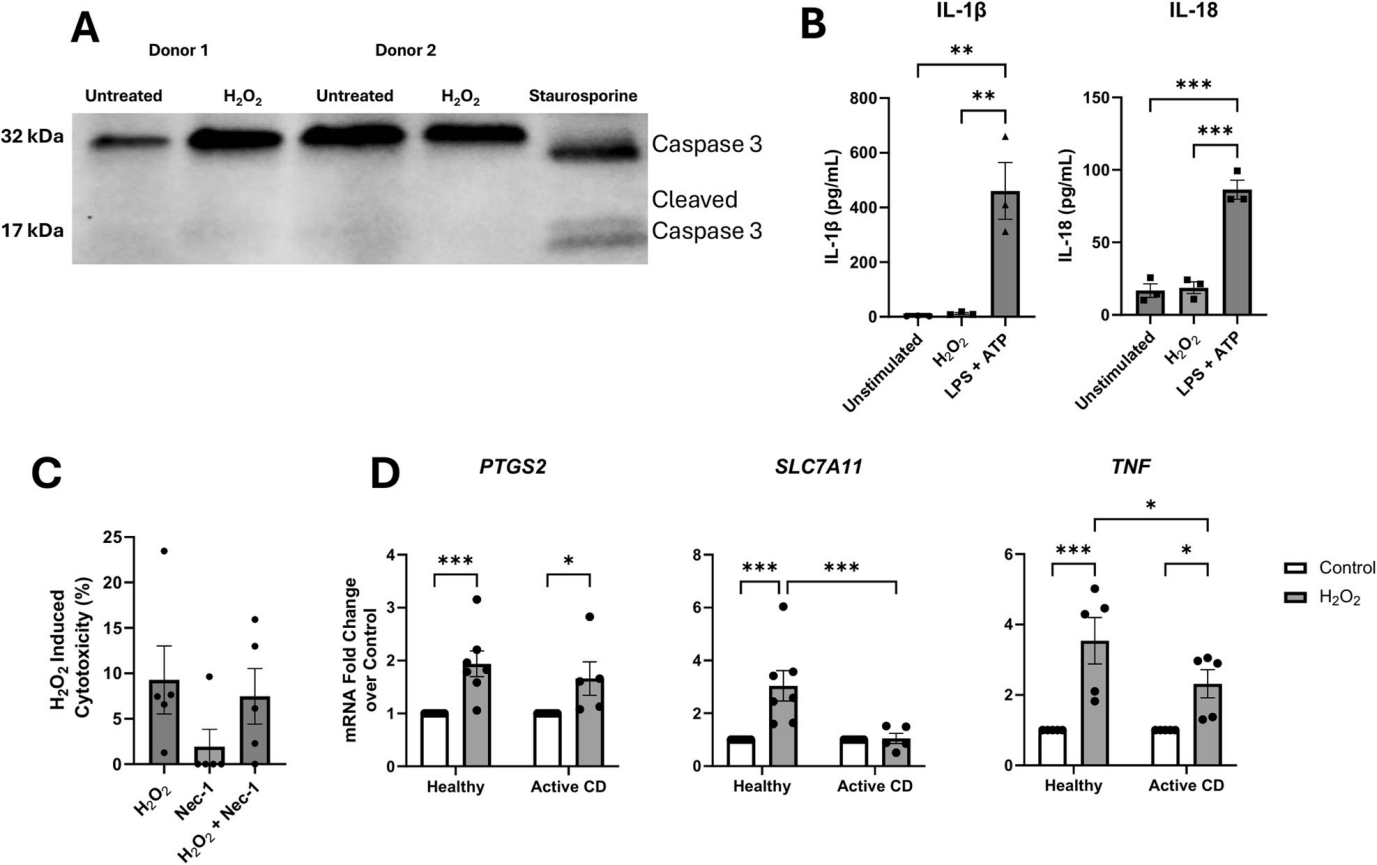
H_2_O_2_ does not induce markers of apoptosis, pyroptosis, or necroptosis. In healthy macrophages (5 × 10^5^ cells), H_2_O_2_ (500 µM, 2 h) does not induce the expression of cleaved caspase-3 (**A**), IL-1β or IL-18 (**B**). Necrostatin-1 (25 µM, 30 min pre-treatment) does not affect the cytotoxicity of H_2_O_2_ of healthy donor macrophages (**C**). As determined by qPCR (**D**), H_2_O_2_ (500 µM, 2 h) induces expression of *PTGS2*, *SLC7A11*, and *TNF* (markers of ferroptosis). Data are means ± SEM (*p < 0.05, ***p < 0.0005).

To assess the mechanism of cell death, we examined cleaved caspase-3 expression. We did not observe an increase in its expression in H_2_O_2_-treated macrophages (Fig. 2A), suggesting death was not via caspase-executed apoptosis. In addition, IL-1β and IL-18, markers of inflammasome activation and pyroptosis, were not increased in the macrophage supernatants (Fig. 2B). Similarly, the use of necrostatin-1 to block necroptosis failed to alleviate the H_2_O_2_ (500 μM, 2 h)-evoked release of LDH (Fig. 2C). However, qPCR revealed increased mRNA expression of *PTGS2, SLC7A11*, and *TNF* in H_2_O_2_-treated macrophages (Fig. 2D), markers associated with ferroptosis [11].

**Fig. 3:**
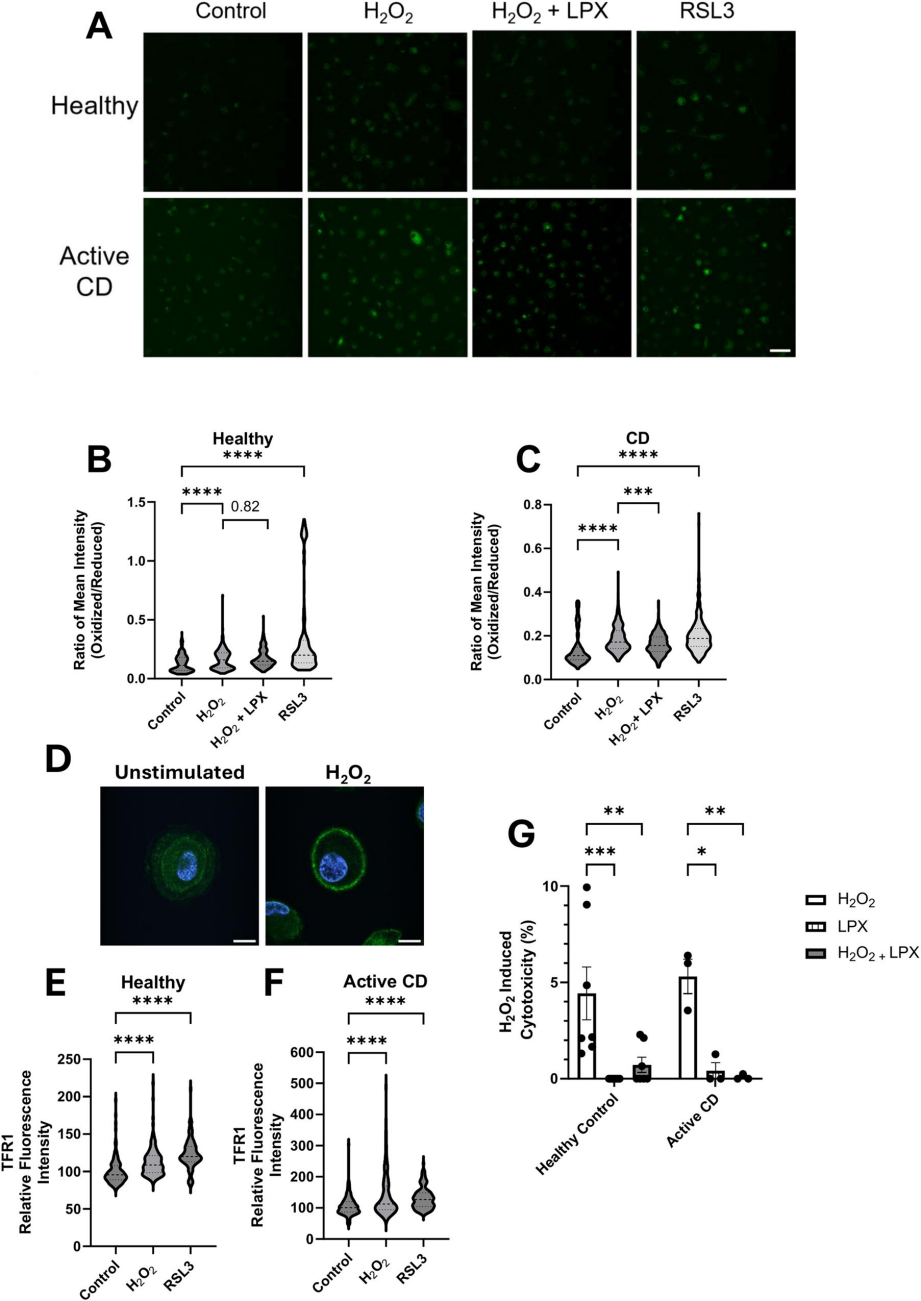
H_2_O_2_ induces macrophage cytotoxicity through ferroptosis. Macrophages (62,500 cells) derived from healthy controls and individuals with active CD were treated with H_2_O_2_ (500 µM, 2 h) ± liporoxstatin-1 (LPX, 10 µM, 30 min pre-treatment) or RSL3 (1 µM, 2 h) and lipid peroxidation and transferrin receptor 1 (TFR1) staining were assessed. H_2_O_2_ induces lipid peroxidation in both healthy macrophages and macrophages derived from patients with active CD (**A**–**C**; 50 cells per donor were analyzed from 4 healthy controls and 5 patients with active CD. Scale bar = 50 µm). H_2_O_2_ and RSL3 induces TFR1 localization to the plasma membrane in healthy (**D**, **E**) and active CD patient (**F**) derived macrophages. (50 cells per donor were analyzed from 4 healthy controls and 3 patients with active CD. Mean fluorescence intensity was normalized to the control. Scale bar = 10 µm. Dashed lines represent the median and dotted lines the quartiles). Liproxstatin-1 blocks the cytotoxic effect of H_2_O_2_ in macrophages derived from healthy controls and patients with active CD (**G**) (*p < 0.05, **p < 0.005, ***p < 0.0005, ****p < 0.0001).

We then used Bodipy-C11 to examine lipid peroxidation, a canonical marker of ferroptosis. After exposure to H_2_O_2_ there was significantly more oxidized Bodipy-C11 in macrophages from healthy donors and individuals with active CD compared to untreated cells indicating increased lipid peroxidation (Fig. 3A–C). RSL3 (1 μM, 2 h) was used as a positive control to induce ferroptosis and lipid peroxidation was increased in both donor groups. Monocyte-derived macrophages from the patients co-treated with liproxstatin 1 (LPX), a radical trapping agent and ferroptosis inhibitor, also displayed significantly reduced amounts of oxidized Bodipy-C11. As a third marker of ferroptosis, the localization and expression of the transferrin receptor 1 (TFR1) was examined [32]. In macrophages treated with H_2_O_2_, there was increased cell surface expression (similar to RSL3 treatment) of TFR1 in healthy and active CD-derived macrophages (Fig. 3D–F). Finally, pre-treatment with liproxstatin 1 virtually abolished the H_2_O_2_-induced cytotoxicity as assessed by LDH release in macrophages from healthy volunteers and individuals with active CD (Fig. 3G).

### Intestinal macrophages from individual with Crohn’s disease have reduced selenoprotein expression

**Fig. 4:**
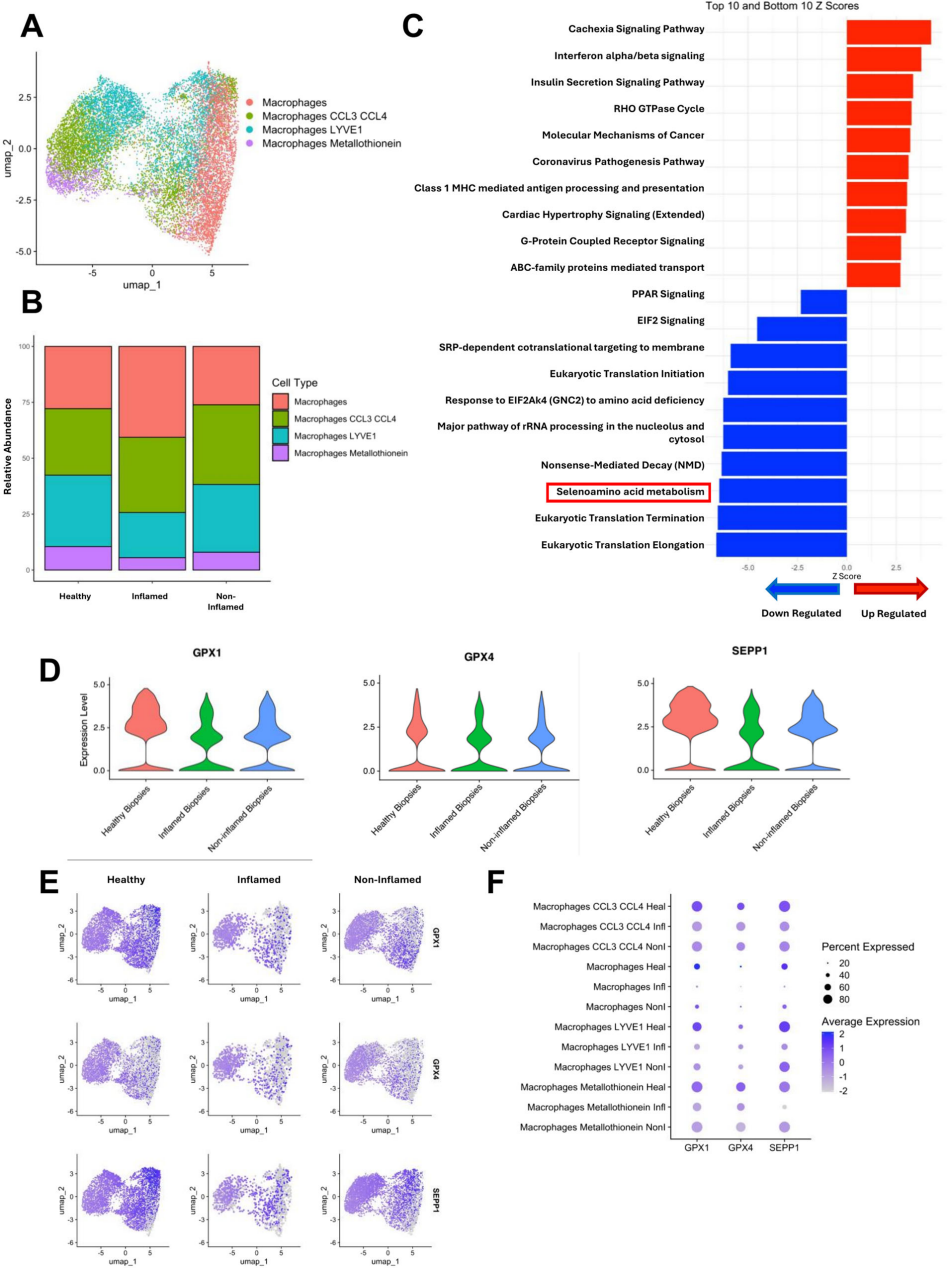
Macrophages from patients with active CD have reduced selenoprotein mRNA expression in situ. Published scRNAseq data (SCP1884) was reanalyzed examining colonic macrophage mRNA expression in healthy biopsies and biopsies from patients with CD. (**A**) UMAP representation of the macrophage clusters. (**B**) Relative abundance of each cluster between groups. Top differentially expression pathways between scRNAseq data from inflamed biopsies from patients with CD compared with healthy biopsies (**C**). The same dataset was used to assess selenoprotein mRNA expression of selenoprotein P (SEPP1), glutathione peroxidase (GPx) 1 and GPx4 in macrophages from healthy biopsies and inflamed and non-inflamed biopsies from patients with CD (**D**) including in the different sub-populations (**E**, **F**). (Heal Healthy, Infl Inflamed, NonI Non-inflamed).

Utilizing published scRNAseq data from inflamed and non-inflamed colonic biopsies from individuals with CD and control biopsies from 71 donors, changes in macrophage populations and gene expression were assessed. The original author annotations were utilized (Supplementary Table 6, Fig. 4A) and revealed increased relative abundance of CCL3^+^CCL4^+^ macrophages and reduced relative abundance of LYVE1^+^ macrophages in inflamed biopsies (Fig. 4B). Furthermore, analysis of the combined macrophage populations revealed 737 differentially expressed genes between the biopsy groups. Gene pathway analysis was conducted and the top ten upregulated and downregulated pathways in the inflamed CD biopsies compared with healthy control biopsies are presented in Fig. 4C. Notably, the selenoamino acid metabolism pathway is downregulated in macrophages in inflamed CD biopsies. Selenium deficiency can occur in individuals with IBD [16, 33, 34]: the full impact of this deficiency on immunity is unknown, specifically with respect to macrophage biology. The mRNA expression of the three most abundantly expressed selenoproteins in macrophages, *SelenoP*, *GPX1*, and *GPX4* [35] were also reduced in tissue macrophages from patients with CD (Fig. 4D) and was seen regardless of the macrophage subpopulation (Fig. 4E, F).

**Fig. 5:**
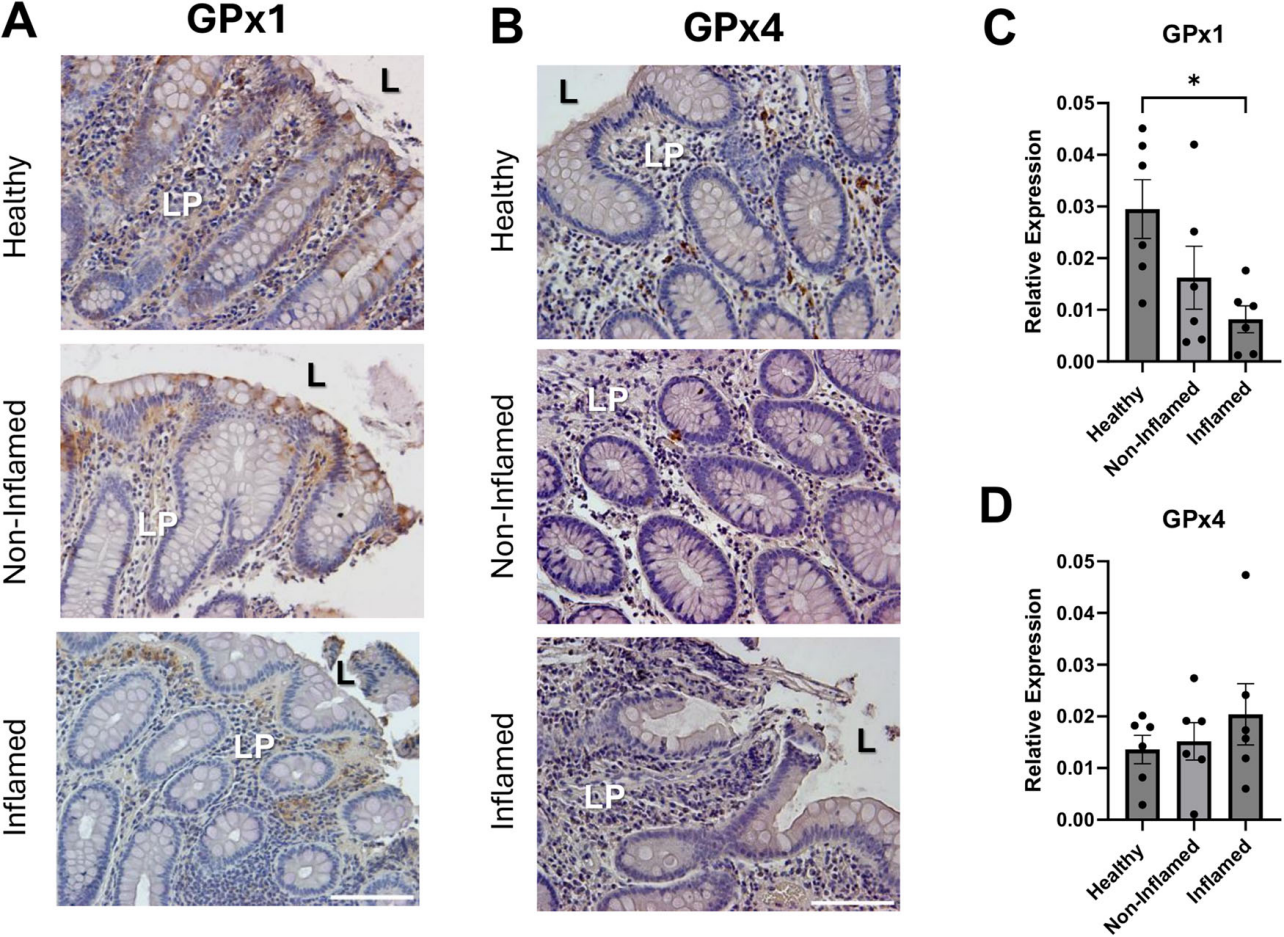
Lamina propria cells in areas of active inflammation have reduced GPx1 expression. Colonic biopsies from healthy individuals and those with Crohn’s disease (inflamed areas and non-inflamed areas) were assessed by immunohistochemistry for GPx1 (**A**, **C**) and GPx4 (**B**, **D**) expression. Representative images show decreased in GPx1-immunoreativity (IR), but not GPx4-IR, in inflamed tissue regions in IBD and as quantified in (**C**) (*n* = 6; L, Lumen; LP, lamina propria. Scale bar = 100 μm; *p < 0.05).

In terms of protein expression, immunolocalization of GPx1 revealed reduced lamina propria expression in biopsies from patients with CD compared to control, that was more marked in biopsies from areas of active inflammation (Fig. 5A, C). While the lamina propria showed reduced GPx1 expression, we cannot unequivocally assign this to reduced expression in macrophages. Differences in GPx4 expression in inflamed biopsies compared to non-inflamed and healthy controls were not observed (Fig. 5B, D).

**Fig. 6:**
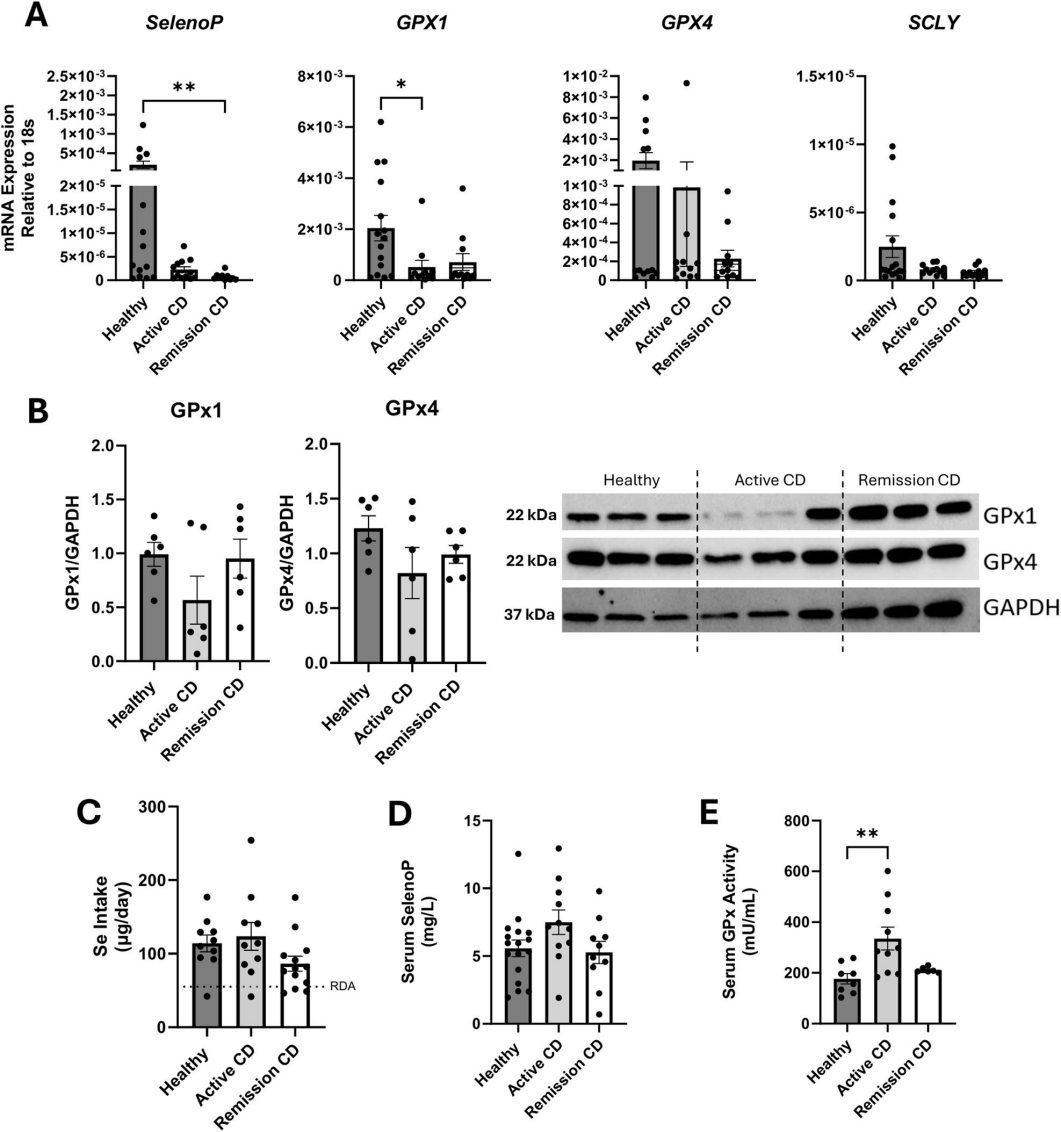
Monocyte-derived macrophages from patients with active CD have reduced selenoprotein expression despite selenium sufficiency. Monocyte-derived macrophages from patients with active CD or individuals in disease remission have reduced selective selenoprotein expression as determined by qPCR (**A**) and immunoblotting (**B**; densitometry (*n* = 3) and a representative blot)) as compared to cells from healthy volunteers. Selenium status was assessed by selenium intake using 24 h food recalls (**C**), serum SelenoP concentration (**D**), and serum GPx activity (**E**) by ELISA and enzymatic activity assay, respectively. Data are means ± SEM (*p < 0.05, **p < 0.005; SCLY, Selenocysteine lyase).

To assess if gut selenoprotein deficiency was mirrored in circulating cells, blood monocyte-derived macrophages were examined. Compared to healthy controls, there was reduced *SelenoP* mRNA expression in macrophages derived from patients with CD in remission (Fig. 6A). Similarly, there was reduced *GPX1* mRNA in macrophages derived from patients with active CD compared with healthy controls. However, there were no differences between the groups in mRNA expression of *GPX4* or the selenium metabolism protein, selenocysteine lyase (*SCLY*). Immunoblot analysis indicated reduced GPx1 protein expression in 4/6 patients with active CD and no differences in GPx4 protein expression (Fig. 6B). Interestingly the two individuals with active CD and high GPx1 expression had H_2_O_2_-induced cytotoxicity within the healthy control range (i.e., 3.5% and 4.4%). Moreover, these two patients were not taking any medications at the time of the blood collection.

To examine whether the differences observed in CD could be due to selenium deficiency, selenium status was assessed. Twenty-four-hour food recall surveys indicated no significant difference in reported selenium intake between the three groups, with all but four individuals consuming more selenium than the recommended daily allowance (RDA) (Fig. 6C). Also, SelenoP concentrations and GPx activity were not reduced in serum from individuals with CD compared to healthy volunteers (Fig. 6D, E). Taken together, there is no evidence in support of selenium deficiency in this cohort of individuals with CD that would explain the reduced selenoprotein expression in the individuals’ monocyte-derived macrophages.

### GPx1 deficiency increases sensitivity to H_2_O_2_ induced ferroptosis

**Fig. 7:**
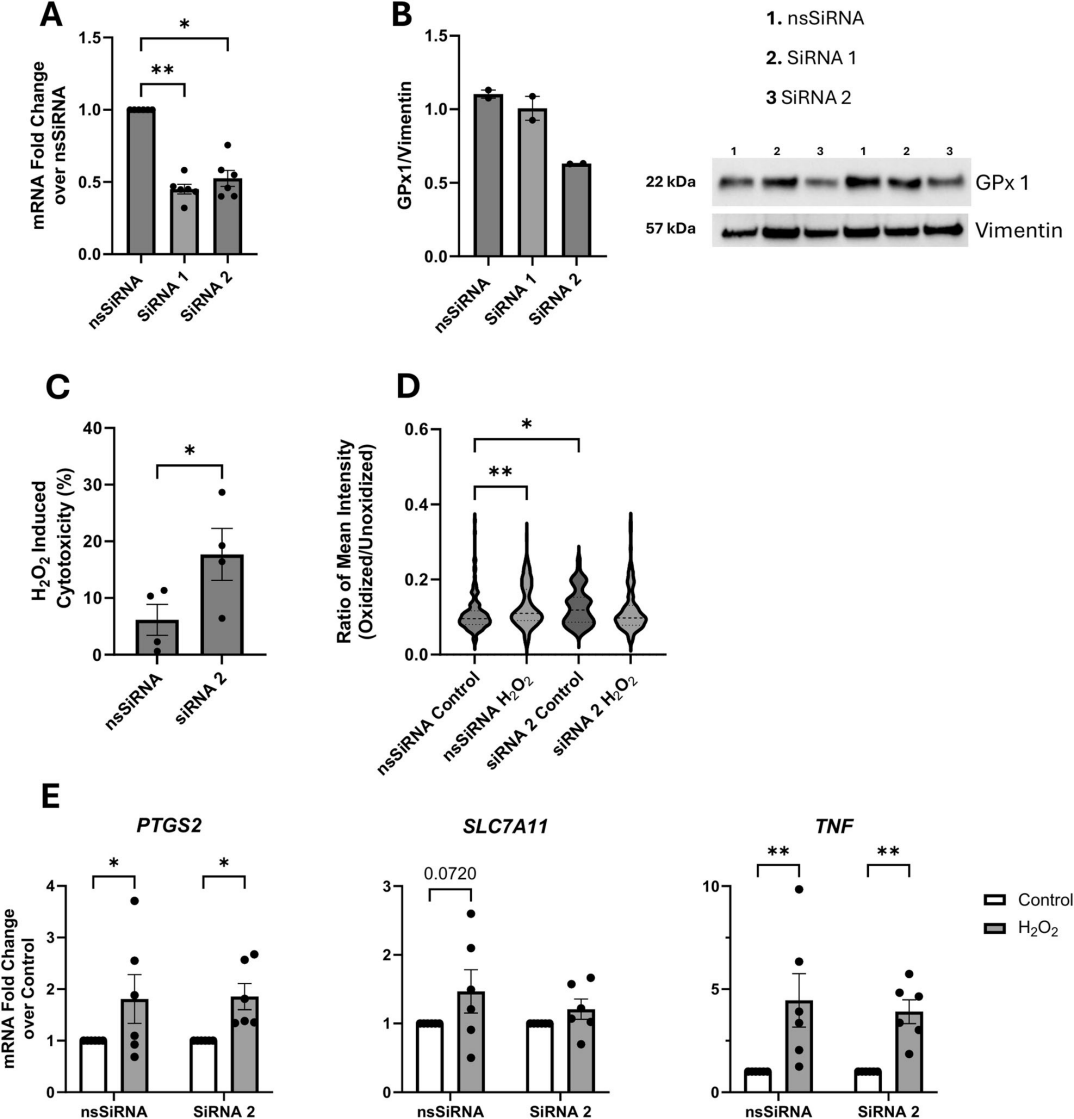
GPx1 knockout increases susceptibility to H_2_O_2_-induced cytotoxicity. Healthy control monocyte-derived macrophages were transfected with siRNA against GPx1 for 48 h and efficiency of knockdown (KD) was assessed by qPCR (**A**) and immunoblotting (**B**) (nsSiRNA, non-specific control SiRNA). GPx1 KD macrophages were treated with H_2_O_2_ (500 μM, 2 h) and cell death (**C**), lipid peroxidation (**D**; 50 cells per donor were analyzed from 3 healthy) and qPCR of ferroptosis markers (**E**) was assessed. Data are means ± SEM. Dashed lines represent the media and dotted lines the quartiles (*p < 0.05, **p < 0.005).

Due to the decrease in GPx1 expression in CD macrophages, we hypothesized that this may contribute to the increased cytotoxicity observed in response to H_2_O_2_. Consequently, macrophages derived from healthy controls were transfected with two different sequences of *GPX1* SiRNA to knockdown (KD) protein expression. Based on mRNA expression both sequences reduced expression to ~45% of the non-silencing control (nsSiRNA; Fig. 7A). However, only cells treated with the SiRNA 2 sequence demonstrated reduced GPx1 protein expression (Fig. 7B). Therefore, SiRNA 2 was used for subsequent experiments.

When treated with H_2_O_2_ (500 μM, 2 h), GPx1-KD macrophages displayed increased cytotoxicity compared to cells treated with the nsSiRNA (Fig. 7C). Furthermore, GPx1-KD macrophages had increased lipid peroxidation compared to control (Fig. 7D): H_2_O_2_ increased lipid peroxidation in nsSiRNA cells, but no further increase was noted in the H_2_O_2_-treated GPx1-KO cell (i.e., where baseline was elevated). Lastly, mRNA expression of both *PTGS2* and *TNF* increased in both nsSiRNA and SiRNA 2 groups after H_2_O_2_ treatment (Fig. 7E), while there was not a significant increase in *SLC7A11* expression mimicking the data observed after treatment of CD macrophages with H_2_O_2_.

## Discussion

With the increasing interest in macrophages as a therapeutic target in IBD [13, 36], it is essential to assess the cell’s susceptibility to stimuli that trigger cell death as this could have implications for the progression of enteric inflammation and vulnerability to infection. In comparison to monocyte-derived macrophages from healthy volunteers, macrophages from patients with active CD were more susceptible to ferroptosis induced by in vitro exposure to H_2_O_2_. By utilizing H_2_O_2_ we mimic the highly oxidative environment of intestinal inflammation and further our understanding of the macrophage’s response to this environment.

The understanding of macrophage function in the gut and in IBD is a complex and evolving topic. For example, the role of macrophages in inflammation has moved away from the dichotomy of pro-inflammatory M1 and anti-inflammatory M2 cells, being replaced by a more nuanced view, encompassing a diversity of heterogeneous macrophage sub-types, likely with specialized functions [36]. The current consensus is that macrophages recruited into the gut in IBD have a greater pro-inflammatory potential than resident macrophages, and this would support the notion that increased sensitivity of the recruited cells to pro-death signals could limit inflammation, while the opposite could be envisaged for resident macrophages. Assessment of CD206^+^CD68^+^ macrophages (a putative tissue-repair cell) revealed reduced numbers in biopsies from patients with IBD [5], but it was not determined if this was due to lack of recruitment, conversion to another phenotype (or loss of CD206 and/or CD68 expression), or increased in situ cell death. In this context, Du et al., noted loss of tissue resident macrophages in colon biopsies from patients with ulcerative colitis, that was accompanied by decreased superoxide dismutase 2 expression (an important antioxidant) in the macrophages [3]. This resulted in increased susceptibility to reactive oxygen species (ROS) and cell death. Therefore, the increased susceptibility to cell death induced by H_2_O_2_ may also contribute to the disappearance of tissue resident macrophages. In contrast, Palmer et al. assessing a panel of six stimuli showed that monocyte-derived macrophages from individuals with CD were more resistant to apoptosis induced by phorbol 12-myristate 13-acetate (PMA) or TNF compared to healthy controls: they suggested that the prolonged presence of these macrophages would contribute to inflammation and limit tissue repair [9]. The discrepancy between this study and the data herein may reflect the heterogeneous nature of CD and disease severity between the patient donors (the current study used monocytes from individuals with mild-to-moderate CD), the mechanisms of action of the various pro-death stimuli, or differences in apoptotic versus non-apoptotic death in macrophages.

Addressing the mechanism of the H_2_O_2_-induced macrophage death, immunoblotting for cleaved caspase-3, measurement of released IL-1β and IL-18, and use of nercostatin-1 did not support caspase-3-dependent apoptosis, inflammasome activation or necroptosis as the underlying cause of the cytotoxicity, respectively. However, the H_2_O_2_-induced increases in *PTGS2*, *SLC7A11*, and *TNF* mRNA, surface expression of the transferrin receptor, and lipid peroxidation are features that accompany ferroptosis—an iron-dependent form of cell death. Use of the pharmacological inducer of ferroptosis, RSL3, elicited a similar level of lipid peroxidation in macrophages compared to H_2_O_2_, while the ferroptosis inhibitor, liproxstatin-1, significantly reduced the H_2_O_2_-induced cytotoxicity in macrophages. Finally, *SLC7A11* mRNA was increased in healthy but not CD-derived macrophages treated with H_2_O_2_, suggesting defective defense mechanisms against ferroptosis due to the role of *SLC7A11* in glutathione synthesis as a substrate for GPx. Collectively, these data implicate ferroptosis as the mode of macrophage death driven by H_2_O_2_ observed in the current study.

There is a lack of data on ferroptosis in IBD, with some recent studies relating to epithelial cells and considerably less on macrophages; the bulk of the current information being generated from murine models of colitis [37]. For example, an enlargement in the labile iron pool in colonic macrophages in the DSS-model of colitis could translate to priming and susceptibility for ferroptotic cell death [38]. Similarly, evidence of increased lipid peroxidation in colonic macrophages from DSS-treated mice or *il-10*^*−/−*^ mice, in which colitis arises spontaneously, is consistent with a propensity for ferroptosis [12, 39, 40]. Furthermore, interventional studies in DSS-colitis suggest targeting ferroptosis could be therapeutic: β-caryophyllene inhibition of macrophage lipid peroxidation and ferroptosis was linked to suppression of inflammation [12], and seliciclib-treated mice—that displayed reduced epithelial ferroptosis—had less severe DSS-induced colitis [41]. While neither agent is specific for ferroptosis, these pre-clinical model studies suggest that a role for ferroptosis in the pathophysiology of IBD is worthy of greater consideration [37].

The role of selenium in ferroptosis has been well described, specifically the selenoprotein GPx4 that is recognized as a canonical suppressor of ferroptosis. Other selenoproteins (e.g., GPx1) have not been implicated in ferroptosis. The data herein suggests that GPx1 deficiency could underlie the increased susceptibility of macrophages from patients with active CD for increased H_2_O_2_-cytotixicity. To test this, GPx1 expression was partially knocked down in macrophages from healthy volunteers and this resulted in increased H_2_O_2_-induced cell death and lipid peroxidation without H_2_O_2_ treatment, implying that GPx1 is important in the homeostatic maintenance of cellular lipid redox. When H_2_O_2_ was added to the GPx1 knock-down macrophages there was no further increase in lipid peroxidation. This was an unexpected finding that requires further investigation but could be a consequence of the increased baseline lipid peroxidation in the GPx1 knock-down cells. Furthermore, it remains unknown whether increasing GPx1 expression in macrophages derived from patients with CD (either genetically or pharmacologically) would reduce their susceptibility to H_2_O_2_-induced cell death to levels comparable to that in healthy macrophages. Future studies addressing this would further advance the awareness of cell death mechanisms and preventative measures in macrophages that could identify new approaches to the management of IBD.

The role of GPx1 in inhibiting ferroptosis may be novel and linked to the nature of the stimuli. For example, when using RSL3—a canonical ferroptosis inducer through inhibition of GPx4—GPx1 cannot compensate for the loss of GPx4 since its main substrates are hydroperoxides whereas GPx4 reduces lipid peroxides [19]. However, when H_2_O_2_ is used, GPx1 plays a major role in reducing H_2_O_2_ to block subsequent downstream events such as lipid peroxidation [19]. These findings are supported by (i) the decreased GPx1 mRNA in intestinal macrophages, and (ii) the decreased GPx1 protein observed in biopsies from inflamed gut regions from individuals with CD, although the immunohistochemistry did not co-stain for macrophage markers. These novel findings are complemented by the demonstration of reduced GPx1 in the colon of mice with DSS-induced colitis [41, 42], murine colitis induced by knockout of GPx1 and GPx2 [43], and genome-wide association studies that note polymorphisms in antioxidant genes, including GPxs in IBD [44, 45].

The mechanism of decreased GPx1 in CD-derived macrophages is unknown and was found to be independent of selenium deficiency. Furthermore, whether this was a consequence of intestinal inflammation, or a primary abnormality has not been determined. Zhao et al. examined the susceptibility of different monocyte subsets to H_2_O_2_-induced cytotoxicity, and reported that CD16^+^ monocytes had increased cell death in response to H_2_O_2_ as well as decreased GPx1 expression [46] (knockdown of GPx1 in CD16^−^ monocytes recapitulated the phenotype). Moreover, there is evidence of increased CD16^+^ monocytes in the blood of individuals with CD and these cells have been implicated as drivers of inflammation as they extravagate the blood vessels and enter the gut tissue [43, 47]. Therefore, the increased susceptibility to H_2_O_2_-induced cell death in macrophages from individual with CD seen in this study may be due to their derivation from the CD16^+^ monocyte population with decreased GPx1 expression. The selective reduction in selenoprotein mRNA and protein in macrophages from CD was observed in bulk culture while assessment of CD16 sub-populations was not conducted. However, it would be intriguing to determine if selenoprotein status correlated with monocyte CD16 expression. In addition, selenoprotein expression could be affected by macrophage polarization state, as proposed for murine cells [48]. However, our pilot studies with IFNγ and IL-4 provided no data in support of this (Supplementary Fig. 1), this remains an area worthy of investigation with clear implications for pro-inflammatory events verse tissue remodeling and repair. As an alternative possibility, the GPx1 deficiency in intestinal macrophages could be due to increased utilization of selenium for selenoprotein production elsewhere as serum GPx activity was increased in patients with active CD.

In the absence of a definitive cause for IBD, and hence a cure, novel and often nuanced aspects of disease pathophysiology continue to be uncovered. Ferroptosis is emerging as a new player in inflammatory disease [49] that may be relevant to IBD [37]. We found that monocyte-derived macrophages from individuals with IBD have increased sensitivity to H_2_O_2_-induced ferroptosis and this was at least partially due to decreased expression of GPx1. The increased susceptibility to cell death could exaggerate inflammation via the release of damage signals (e.g., ATP, High mobility group box 1 protein (HMGB1)) or alternatively, ferroptosis of CD16^+^ monocytes and/or macrophages that are reputedly more pro-inflammatory than intestinal macrophages could serve to limit inflammation. Thus, while identifying ferroptosis as an aspect of the macrophage in CD, much remains to be done to understand the physiological and pathophysiological implications of death pathways in myeloid cells. Future studies should examine if the increased susceptibility to ferroptosis occurs with other stimuli and whether the consequences of cell death exacerbate or limit inflammation. Finally, whether the decreased GPx1 in CD-derived macrophages is a result of intestinal inflammation, increased utilization of selenium elsewhere in the body or a polymorphism in the *GPX1* gene should be tested.

## Supplementary information

Supplemental Tables

Original Western Blots

## Data Availability

All data generated or analyzed during this study are included in this published article [and its supplementary information files].
