# Supplementary material for: GPx1 deficiency confers increased susceptibility to ferroptosis in macrophages from individuals with active Crohn’s disease
Source: Cell Death Dis. 2024 Dec 18;15(12):903. doi: 10.1038/s41419-024-07289-y (PMC11655851; doi:10.1038/s41419-024-07289-y)
Supplement: Supplementary file 1 — Supplemental Tables [file 41419_2024_7289_MOESM1_ESM.docx]

**Table S1** Active Crohn’s Disease (CD) Patient Inclusion and Exclusion Criteria

| Inclusion Criteria |
| --- |
| - Adult patients≥18 years of age - Clinical diagnosis of luminal ileal, ileo-colonic, or colonic CD - Harvey-Bradshaw Index <16 at the time of recruitment - Biomarker evidence of inflammation at enrolment, defined as one or more of the following: - FCP≥250 µg/g within 4–6 weeks prior to recruitment or CRP > 8 mg/L or endoscopic or cross-sectional evidence of disease activity per standard measures - Stable biologic, immunosuppressant or aminosalicylate dosing for 4 weeks prior to study enrolment - Able to provide informed consent |
| Exclusion Criteria |
| - Harvey-Bradshaw Index≥16 at the time of recruitment - Upper gastrointestinal tract CD - Evidence of active perianal or fistulising disease - >One small bowel resection - Significant chronic disorders such as cardiac disease, renal failure, and active pulmonary disease - Any psychiatric or neurocognitive disorder that would limit ability to participate - Use of a laxative within 1 month prior to recruitment - Use of antibiotics within 2 months prior to recruitment - Presence of an ostomy - Any change (i.e., starting, stopping, or changing dose) in medication for CD, apart from corticosteroids, within 4 weeks prior to recruitment - COVID-19 within 8 weeks prior to recruitment or during the study - Pregnant or breast feeding |

Table S2 Crohn’s Disease Patient in Remission (CD) Inclusion and Exclusion Criteria

| Inclusion Criteria |
| --- |
| - Adult patients≥18 years of age - Harvey-Bradshaw Index<5 at the time of recruitment. - Clinical diagnosis of luminal ileal, ileo-colonic, or colonic CD |
| Exclusion Criteria |
| - Upper gastrointestinal tract CD - Evidence of active perianal or fistulising disease - >One small bowel resection - Any psychiatric or neurocognitive disorder that would limit ability to participate |

Table S3 Characteristics of Patients Used in Macrophage Analysis

| **Patient ID** | **Age** | **Sex** | **Smoking Status** | **Montreal Classification of Disease Location** | **Medication** | **Year of Diagnosis** |
| --- | --- | --- | --- | --- | --- | --- |
| **Active Crohn’s disease** | | | | | | |
| TDI-027 | 25 | F | Non-Smoker | L2 | 5-ASA, Escitalopram | 2021 |
| TDI-028 | 45 | M | Non-Smoker | L2 | None | 2018 |
| TDI-030 | 19 | M | Non-Smoker | L1 | None | 2021 |
| TDI-032 | 29 | M | Non-Smoker | L2 | None | 2022 |
| TDI-034 | 31 | F | Non-Smoker | L3 | None | 2021 |
| TDI-036 | 32 | M | Non-Smoker | L3 | Budesonide | 2022 |
| TDI-037 | 55 | M | Current Smoker | L2 | Anti-TNF | 2020 |
| TDI-038 | 58 | F | Non-Smoker | L2 | Prednisone | 2008 |
| TDI-040 | 37 | M | Non-Smoker | L3 | 5-ASA | 2003 |
| TDI-041 | 34 | F | Non-Smoker | L3 | None | 2008 |
| TDI-042 | 58 | M | Non-Smoker | L1 | 5-ASA | 1988 |
| TDI-049 | 58 | M | Non-Smoker |  | None |  |
| TDI-058 | 58 | M | Non-Smoker |  | None |  |
| CDF-003 | 33 | M | Non-Smoker |  | Azathioprine, Anti-IL-12/IL-23 |  |
| CDF-004 | 67 | F | Non-Smoker |  | Amgevita, Alendronate Sodium Monohydrate, Valtrex |  |
| BC99 | 66 | F | Non-Smoker |  | None | 1990 |
| BC100 | 56 | M | Non-Smoker |  | Anti-TNF | 2022 |
| BC101 | 62 | F | Non-Smoker |  | Anti-TNF | 2023 |
| BC112 | 69 | M | Non-Smoker |  | Anti-TNF | 2010 |
| BC114 | 20 | M | Non-Smoker |  | Anti-IL-23 | 2021 |
| **Crohn’s disease in Remission** | | | | | | |
| PFZ-024 | 51 | F | Non-Smoker | N/A | Azathioprine | 1998 |
| PFZ-026 | 52 | F | Non-Smoker | N/A | Anti-TNF | 2017 |
| PFZ-027 | 31 | M | Current Smoker | L2 | None | 2022 |
| PFZ-029 | 47 | M | Non-Smoker | L2 | None | 2014 |
| PFZ-030 | 45 | F | Non-Smoker | N/A | Anti-TNF | 1998 |
| PFZ-031 | 33 | F | Non-Smoker | N/A | None | 2013 |
| PFZ-032 | 35 | F | Non-Smoker | N/A | Anti-IL-12/IL-23 | 2014 |
| PFZ-034 | 22 | M | Non-Smoker | N/A | Anti-TNF | 2021 |
| PFZ-035 | 55 | F | Non-Smoker | N/A | None | 1989 |
| PFZ-036 | 61 | M | Non-Smoker | N/A | Anti-IL-12/IL-23 | 1988 |
| PFZ-037 | 32 | M | Non-Smoker | N/A | Anti-α4β7 | 2016 |
| PFZ-038 | 59 | F | Non-Smoker | N/A | Anti-TNF | 2006 |
| PFZ-039 | 46 | M | Non-Smoker | N/A | Anti-TNF | 1998 |
| PFZ-063 | 52 | M | Non-Smoker | N/A | 5-ASA |  |
| PFZ-064 | 37 | F | Non-Smoker | N/A | Anti-TNF |  |
| PFZ-065 | 25 | F | Non-Smoker | N/A | Anti-TNF |  |

**Table S4** Primary antibodies used for immunoblotting

| **Protein** | **Host Species** | **Stock Concentration** | **Dilution** | **Source** | **Catalog Number** |
| --- | --- | --- | --- | --- | --- |
| GPx1 | Rabbit | 1 mg/mL | 1:1000 | Abcam | Ab22604 |
| GPx4 | Rabbit | 0.485 mg/mL | 1:1000 | Abcam | Ab125066 |
| GAPDH | Mouse | 2 mg/mL | 1:2500 | Abcam | Ab8245 |
| Total Caspase 3 | Rabbit |  | 1:1000 | Cell Signalling | 9662 |
| Cleaved Caspase 3 (ASP175) (5A1E) | Rabbit |  | 1:1000 | Cell Signalling | 8664S |

Table S5 Primer Sequences for qPCR for Human Genes

| **Name/Function** | **Gene** | **Reference Sequence** | **Forward Primer** | **Reverse Primer** |
| --- | --- | --- | --- | --- |
| Structural Ribosomal RNA | *18s* | NR_003286.2 | ATGGCCGTTCTTAGTTGGTG | CGCTGAGCCAGTCAGTGTAG |
| Selenoprotein P | *SelenoP* | NM_005410.4 | CGAGATATGCCAGCAAGTGA | GGTGATTGCAGACCCTGTTT |
| Glutathione Peroxidase 1 | *GPx 1* | NM_000581.4 | ATTCCCTCAAGTACGTCCGG | GGAGACCAGGTGATGAGCTT |
| Glutathione Peroxidase 4 | *GPx 4* | NM_002085.5 | GCCAGGGAGTAACGAAGAGA | CAGCCGTTCTTGTCGATGAG |
| Cystine/Glutamate Antiporter | *SLC7A11* | NM_014331.4 | TCCGATCTTTGTTGCCCTCT | GACTGTCGAGGTCTCCAGAG |
| Cyclooxygenase 2 | *PTGS2* | NM_000963.4 | TACCCTCCTCAAGTCCCTGA | ACTGCTCATCACCCCATTCA |

**Table S6** scRNAseq Macrophage Subsets

| **Macrophage Subset** | **Markers** |
| --- | --- |
| Macrophages | CD163, C1QC, C1QA, C1QB |
| Macrophages CCL3+ CCL4+ | CCL3, CCL4, DAB2, A2M |
| Macrophages LYVE1+ | LYVE1, F13A1, CCL18 |
| Macrophages Metallothionein | MT1G, MT1X, MT2A, MT1H, MT1E, MT1F, MT1M |
