# Supplementary material for: GPx1 deficiency confers increased susceptibility to ferroptosis in macrophages from individuals with active Crohn’s disease
Source: Cell Death Dis. 2024 Dec 18;15(12):903. doi: 10.1038/s41419-024-07289-y (PMC11655851; doi:10.1038/s41419-024-07289-y)

Supplementary Figure 1

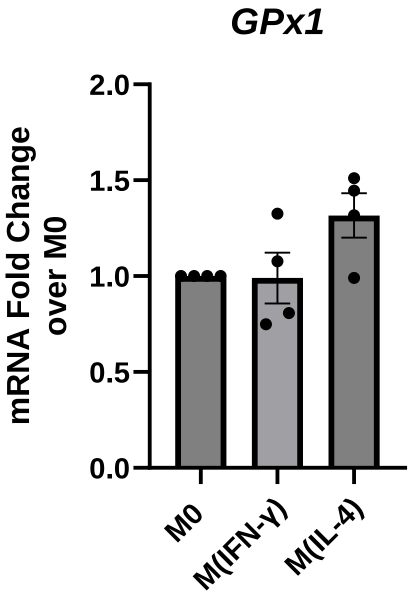

Supplementary Figure 1
Monocyte-derived macrophages ($1.25x10^5$cells) were stimulated with either IFN-γ (10 ng/mL) or IL-4 (10 ng/mL) for 48h and *GPx1* mRNA expression was assessed by qPCR. Data are means ± SEM.

Supplementary Figure 2
Figure 2

Caspase 3

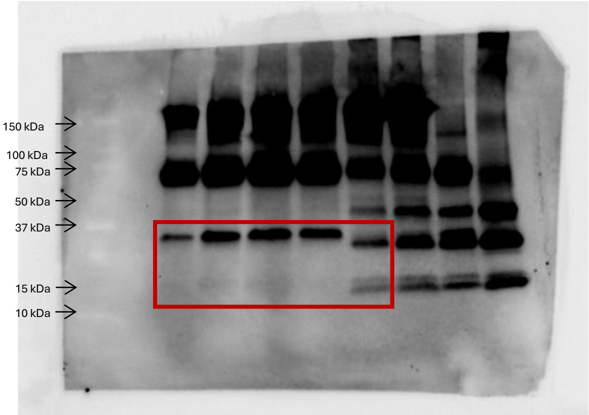

Supplementary Figure 3
Figure 6

GPx1

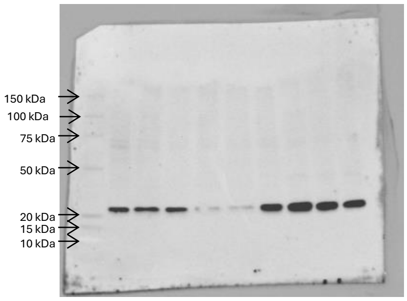

GAPDH

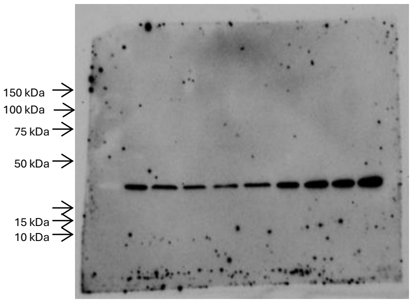

GPx4

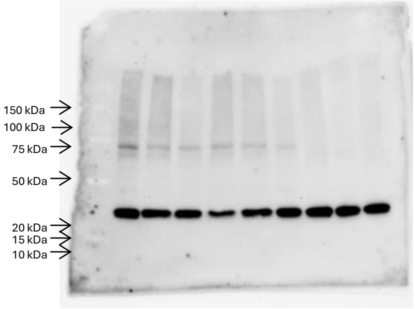

Supplementary Figure 4
Figure 7

GPx1

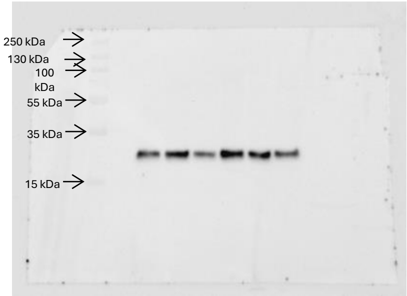

Vimentin

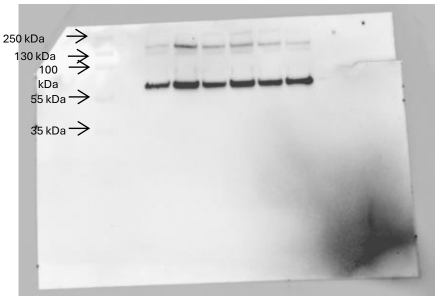

Supplement: Supplementary file 2 — Original Western Blots [file 41419_2024_7289_MOESM2_ESM.pdf]
